# A visualized and bibliometric analysis of nursing research during the COVID-19 pandemic

**DOI:** 10.1097/MD.0000000000039245

**Published:** 2024-08-09

**Authors:** Lu Yang, Yao Wang, Xin Mu, Yanbiao Liao

**Affiliations:** a Department of Cardiology, West China Hospital, Sichuan University/West China School of Nursing, Sichuan University, Chengdu, China; b Department of Cardiology, West China Hospital, Sichuan University, Chengdu, China.

**Keywords:** bibliometric analysis, CiteSpace, COVID-19, nursing research, research trends

## Abstract

**Background:**

The global spread of Coronavirus disease 2019 (COVID-19) has been increasing since December 2019. A total of 8460 publications were obtained from the Web of Science Core Collection from 2019 to 2023, providing insights into the progress of nursing research throughout the COVID-19 pandemic.

**Methods:**

Bibliometric analysis was conducted on these articles using CiteSpace. The analysis focused on examining the distribution of these publications in terms of space and time, distribution of authors, subject categories, distribution of topics, and cited references.

**Results:**

These results may be explained from 3 perspectives. Initially, the number of yearly publications on nursing research consistently increased during the COVID-19 pandemic. Furthermore, a co-occurrence analysis of the countries and the authors revealed that certain countries, including the United States, China, and England, have successfully implemented organized and standardized nursing models. These countries also have well-developed and established nursing research systems. Notably, academic communities in specific regions, such as the team led by MD Stefan Gravenstein, Mor Vincent, and White Elizabeth at Brown University in the United States, have emerged as leaders in this field. Furthermore, examining the papers’ subject categories and topic distribution indicate that nursing during the COVID-19 pandemic has been predominantly interdisciplinary, encompassing various disciplines such as clinical medicine, essential medicine, psychology, public health management, and even telematics science.

**Conclusion subsectiongs:**

Our study provided valuable insights into acquiring knowledge on nursing research during the COVID-19 pandemic, pinpointed possible partners for researchers interested in nursing, and uncovered prevalent research patterns and popular subjects.

## 1. Introduction

The global transmission of COVID-19 has exhibited significant acceleration since December 2019. The rapid and extensive spread of the virus compelled the World Health Organization (WHO) to designate COVID-19 as a “World Health Emergency” in late January 2020, followed by its classification as a Global Pandemic on March 11, 2020.^[1]^ On May 5, 2023, WHO declared COVID-19 no longer classified as a “public health emergency of international concern” (PHEIC).^[2]^ As of November 19, 2023, more than 772 million confirmed illnesses and over 6 million fatalities have been documented worldwide.^[3]^ During the PHEIC, many patients were under severe pressure on hospital resources^[4]^; healthcare workers, especially nurses, at the forefront of this battle played a crucial role in supporting recovery and were impacted the most.^[5,6]^

Amidst the dynamic progression of the COVID-19 outbreak, fluctuating data, and continuous discovery of new research results, nursing research during the pandemic has been remarkably comprehensive.^[7,8]^ It covers a wide range of areas, including epidemic control, post-outbreak assistance, and epidemic prevention.^[9–11]^ In particular, the challenges faced by nurses in caring for patients with COVID-19,^[12]^ the implementation of infection control measures, the use of personal protective equipment, the psychological and emotional impact of the pandemic on nurses,^[13,14]^ the role of nursing in public health intervention during the pandemic, the latest evidence of nursing intervention measures to prevent the spread of COVID-19,^[15]^ the role of nursing leaders in crisis management,^[16]^ and the development of education plans to prepare nurses for the challenges brought by the epidemic.^[17]^ Unsurprisingly, scientists and health professionals have disseminated a large body of correspondence and publications to address nursing,^[18,19]^ medical, and economic^[20]^ research problems during the COVID-19 pandemic. Nevertheless, the rapid distribution of several studies within a short timeframe, exhibiting considerable variation in their quality and reliability, has resulted in an overwhelming amount of information. This further emphasizes the necessity of assessing and consolidating more trustworthy studies to comprehend the complexities of the issue.^[21]^ Nevertheless, researchers face challenges in comprehending the present condition and emerging patterns from a multitude of qualitative and quantitative studies from a singular viewpoint.^[22]^ Some academic research has focused on the knowledge structure and progress of research on pediatrics,^[23]^ neurology,^[24]^ urology,^[25]^ and health literacy^[22]^ during the COVID-19 pandemic using graphical and bibliometric analysis. This work aims to utilize graphical and bibliometric analyses, specifically with the assistance of CiteSpace,^[26]^ to showcase the knowledge structures and advancements in nursing research throughout the COVID-19 pandemic. CiteSpace is a popular tool for visualizing and analyzing academic literature. It has the functions of co-occurrence analysis, network visualization, time zone analysis, keyword clustering, and sudden detection, which helps researchers find nursing research models and trends related to COVID-19, identify key participants in nursing research during the epidemic and their cooperation, show how specific topics emerge or evolve, and determine the theme structure, new trends and hot spots of nursing research in the context of COVID-19.

Zhang et al^[27]^ summarized and visualized the research on COVID-19 and nursing as of March 24, 2022, through bibliometric analysis. On May 5, 2023, WHO announced that COVID-19 would no longer be listed as PHEIC. This study selected the CiteSpace method to conduct a bibliometric analysis of nursing research during the COVID-19 pandemic to supplement the inadequacy and outdatedness of existing literature. The remainder of this paper is organized as follows. The next section outlines the materials and procedures employed to obtain bibliometric data. The subsequent section presents an in-depth examination of the visualization and analysis of the distribution of scientific output, nations, institutions, authors, keywords, and highly cited publications. The concluding section encompasses primary discoveries, constraints, and prospective investigations. The main purpose of this study is to provide valuable insights for obtaining nursing research knowledge during the COVID-19 pandemic, identify possible partners for researchers interested in nursing, and reveal the popular research model and themes.

## 2. Materials and methods

### 2.1. Data source and search

Currently, various databases are available for data collection and bibliometric analysis. Web of Science databases are favored because they are widely used in bibliometric research, covering a large number of journals in the fields of medicine and social sciences and screening a large number of journals with high influencing factors, making it easy to obtain data for analysis.

This study required 2 steps for CiteSpace data collection. The first selected the Science Citation Index Expanded and the Social Science Citation Index of the Web of Science Core Collection Database (https://www.webofscience.com). The second step was to design an accurate retrieval strategy. This study used topic search to retrieve datasets, an ideal retrieval model, as it can describe article content from the perspectives of title, abstract, and keywords. The search method employed was TS = (“COVID-19” OR “2019 Novel Coronavirus Disease” OR “2019-nCoV Disease” OR “SARS-CoV-2” OR “SARS-CoV-2 Infection”) AND TS = nursing AND LA = English. The search was limited to journal articles, with no restrictions on the journals and authors. A cumulative count of 9464 outcomes was identified, spanning 2019 to 2023 (retrieved on July 11, 2023). Subsequently, publications were eliminated based on document type, resulting in a final database comprising only 8460 items, including 7823 articles and 637 reviews. All data, including titles, authors, abstracts, keywords, cited references, and other relevant information, were loaded into CiteSpace6.2. R4: Java-based knowledge mapping tool. This tool is utilized to visually represent patterns and trends in scientific literature.^[26,28]^

### 2.2. Bibliometric analysis methods

CiteSpace is a publicly accessible Java-based program. Its purpose is to study and display trends and patterns in the scientific literature. It presents the structure and distribution of scientific information.^[29,30]^ The concentration is identifying crucial junctures in advancing a topic or domain, namely intellectual milestones and significant moments.^[26]^ After importing literature into CiteSpace, the initial task was to perform data cleansing. If there are no duplicates, the original data can be utilized as is; otherwise, duplicate entries should be eliminated prior to further analysis. After importing the data from this study into CiteSpace, data cleaning was performed, and it was found that there were no duplicate data. CiteSpace facilitates examining various networks formed from scientific publications, encompassing cooperation, co-occurrence, and co-citation networks. Additionally, it enables the analysis of networks consisting of hybrid node types such as words, authors, and nations. The results are presented in graphic graphs, with nodes representing the research topics. The size of each node corresponds to the frequency of its appearance or citations, indicating its significance level. Connections between nodes represent either co-occurrence or co-citation, with the thickness of the line indicating the intensity of these associations: a thicker line signifies a stronger link. The hue or tint of the node and link color signify the sequential arrangement of an item.^[30]^ Furthermore, cluster analysis is an effective method for rapidly studying the knowledge networks in CiteSpace. To be more precise, words in the literature were categorized according to their resemblance. Each cluster was evaluated using a specialized algorithm to assign scores. The phrase with the highest score in each cluster is chosen as the representative, also known as the cluster label. Cluster size corresponds to the number of items that have been clustered together. CiteSpace assigns ID #0 to the largest cluster, ID #1 to the second largest cluster, and so on. Based on the scale of the data in this study and referring to the instructions provided by the CiteSpace author,^[29]^ all default operating parameters were used in this analysis. The parameters of CiteSpace were set as follows: year per slice for one year; term source including Title, Abstract, Author Keywords, and Keywords Plus; and top 50 levels as a threshold that are cited or most frequent in each slice. Node types and visualization were set according to specific conditions.

## 3. Results and discussion

### 3.1. Annual quantitative distribution of publications

**Figure 1.**
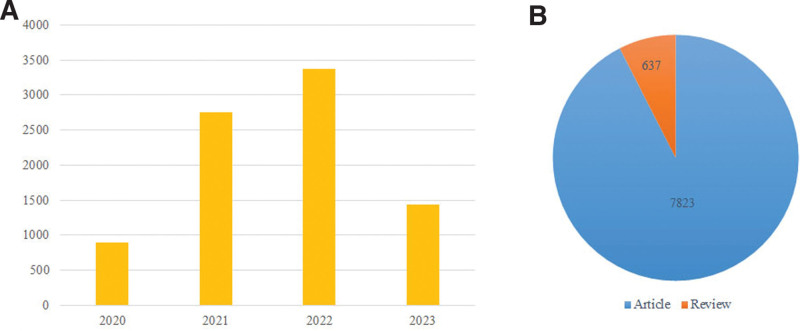
Yearly quantity and document type of publications about nursing research during the COVID-19 pandemic. (A) Annual quantitative distribution. (B) Document type.

The yearly quantity of published papers serves as a measure of the rate at which topic knowledge advances and is a notable indication for analyzing trends in the area.^[31]^ Figure 1 presents the annual volume and document category of research articles on nursing research during the COVID-19 pandemic. A total of 8460 publications were retrieved, including 897 articles published in 2020, 2756 articles published in 2021, 3366 articles published in 2022, and 1441 articles published in 2023 (retrieved on July 11). It can be observed that the number of literatures that satisfied the inclusion criterion has quadrupled annually from 2020 to 2022 (Fig. 1A). The field of nursing research has progressed significantly since 2021 during the COVID-19 pandemic, indicating growing recognition and focus on nursing research. COVID-19 epidemic was increasing and becoming popular worldwide. As of July 11, 2023, 1441 articles were published in 2023. Considering factors such as the end of COVID-19 announced by WHO, the reduction in global detection and reporting, and the delay in publishing the final literature. Moreover, articles comprised approximately 92.5% of the various categories of documents (Fig. 1B), suggesting a significant focus on clinical practice. This shows that original studies, such as case reports and clinical trials, were the primary focus of nursing research during the COVID-19 pandemic.

### 3.2. Country ranking and coauthor analysis

**Figure 2.**
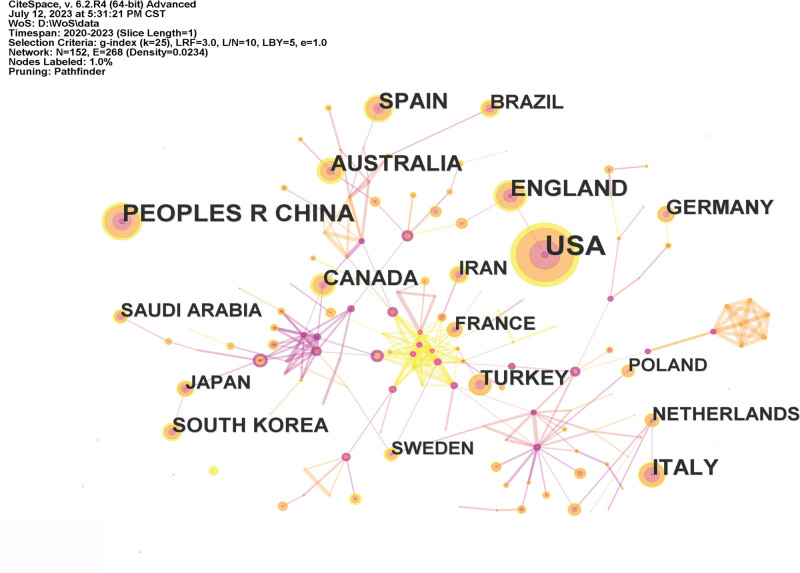
Network of countries of publications about nursing research during the COVID-19 pandemic.

**Figure 3.**
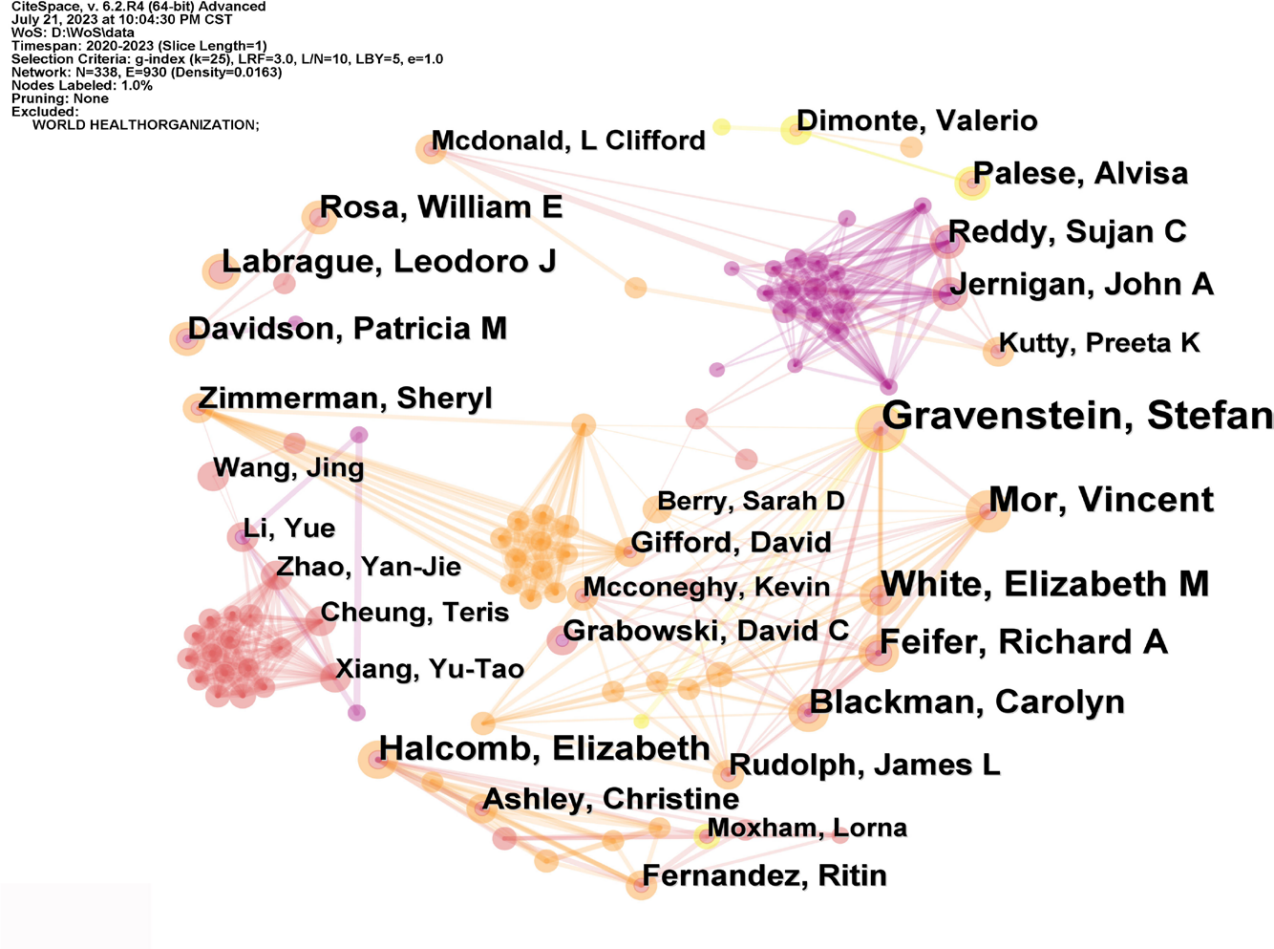
Network of authors of publications about nursing research during the COVID-19 pandemic.

Visualized knowledge mapping facilitates the identification of significant research teams and possible collaborators, enabling researchers to develop collaborative partnerships.^[32]^ An examination of the geographical distribution of these publications indicates that the United States, China, and England are the most prominent countries for coauthored papers and have the highest number of articles written in collaboration (Table 1). This suggests that these countries have made significant contributions to nursing research during the COVID-19 pandemic, in terms of both quantity and quality (Fig. 2). These nations were pioneers in prioritizing and executing nursing research during the COVID-19 pandemic. These countries have established many academic communities that are actively engaged in this topic. Stefan Gravenstein, a medical doctor and public health expert from the Brown University School of Public Health, is considered a prominent person in this field. Gravenstein’s research focuses on nursing homes and healthcare professionals. He is a very productive coauthor who has made a significant contribution to the area of nursing research during the COVID-19 pandemic, as seen in Table 2. Mor Vincent and White Elizabeth M are affiliated with Brown University School of Public Health. Feifer Richard A and Blackman Carolyn are employees of Genesis Healthcare Inc., a prominent post-acute care provider in the United States. Genesis Healthcare Inc. operates almost 250 skilled nursing institutions and senior living communities across 22 states. The researchers discovered characteristics that increase the chances of death among nursing home residents in the USA who have contracted COVID-19. Additionally, advancements in medical treatment and supportive care provided in hospitals have contributed to a decrease in mortality rates.^[33,34]^ Elizabeth Halcomb, a researcher from the University of Wollongong, is dedicated to addressing the immediate support requirements of primary healthcare nurses throughout the COVID-19 pandemic. She proposed that it is imperative to tackle many crucial concerns pertaining to personal health and safety, care quality, and job security in order to provide sufficient support to primary healthcare nurses during the COVID-19 pandemic.^[35]^ Labrague Leodoro from Sultan Qaboos University is now studying the effects of the COVID-19 pandemic on mental health and attitudes towards psychological care among medical and nursing workers.^[36,37]^ Upon observation, it is evident that there are a greater number of connections among the nodes representing authors compared to the nodes representing nations in Figure 3. This suggests that the level of engagement and collaboration between the countries in the area may be lacking. Partnerships between academic teams in each nation continue to be predominantly characterized by cooperation.

**Table 1:** Top 10 productive countries of publications about nursing research during the COVID-19 pandemic.

Rank	Country	Publication frequency
1	USA	2559
2	Peoples R China	958
3	England	638
4	Spain	541
5	Australia	479
6	Italy	463
7	Canada	412
8	Turkey	338
9	South Korea	253
10	Germany	248

**Table 2:** Top 10 productive authors of publications about nursing research during the COVID-19 pandemic.

Rank	Author	Publication frequency	Centrality
1	Gravenstein, Stefan	27	0.01
2	Mor, Vincent	18	0.01
3	White, Elizabeth M	17	0
4	Feifer, Richard A	15	0
5	Halcomb, Elizabeth	15	0
6	Blackman, Carolyn	14	0
7	Labrague, Leodoro J	13	0
8	Palese, Alvisa	12	0
9	Reddy, Sujan C	12	0.02
10	Rosa, William E	11	0

### 3.3. Institutions analysis

**Figure 4.**
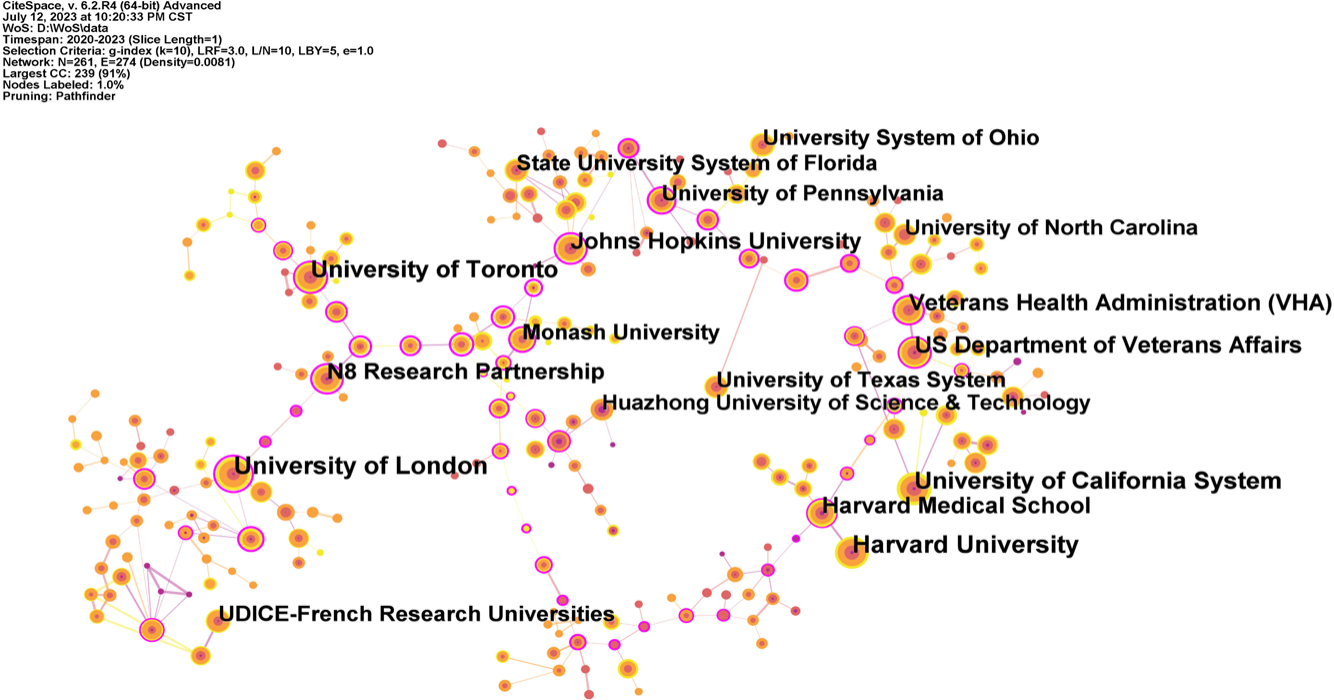
Network of institutions of publications about nursing research during the COVID-19 pandemic. UDICE, VHA = Veterans Health Administration.

Table 3 displays the 10 leading establishments in the domain of nursing research throughout the COVID-19 pandemic. The collaboration network graph consists of the top 10 core academic institutions, with 6 being American institutes, 2 being English institutes, 1 being a Canadian institute, and 1 being a French institute. In terms of node size and publications, the University of London (193), Harvard University (187), and the University of California System (181) are the most significant academic outputs among these institutes, following the University of Toronto (149), the US Department of Veterans Affairs (134), and Johns Hopkins University (134). Institutes that have published more than 100 but less than 130 papers are anticipated to make significant and exceptional contributions to nursing research in the context of COVID-19. These institutes included the Veterans Health Administration (128), Harvard Medical School (127), N8 Research Partnership (119), and UDICE-French Research Universities (101). Figure 4 illustrates the collaboration between key institutions, with particular emphasis on cooperation between domestic entities. For instance, it highlights the close relationship between Harvard University and Harvard Medical School, the connection between the US Department of Veterans Affairs and the Veterans Health Administration, and the linkages between the University of London and the N8 Research Partnership. The connection strength between distinct institutions is directly proportional to the thickness of the connecting line and inversely proportional otherwise.

**Table 3:** Top 10 productive institutions of publications about nursing research during the COVID-19 pandemic.

Rank	Institution	Publications	Centrality
1	University of London	193	0.61
2	Harvard University	187	0
3	University of California System	181	0.08
4	University of Toronto	149	0.24
5	US Department of Veterans Affairs	134	0.3
6	Johns Hopkins University	134	0.77
7	Veterans Health Administration	128	0.45
8	Harvard Medical School	127	0.19
9	N8 Research Partnership	119	0.64
10	UDICE-French Research Universities	101	0

### 3.4. Topic distribution analysis based on keywords

**Figure 5.**
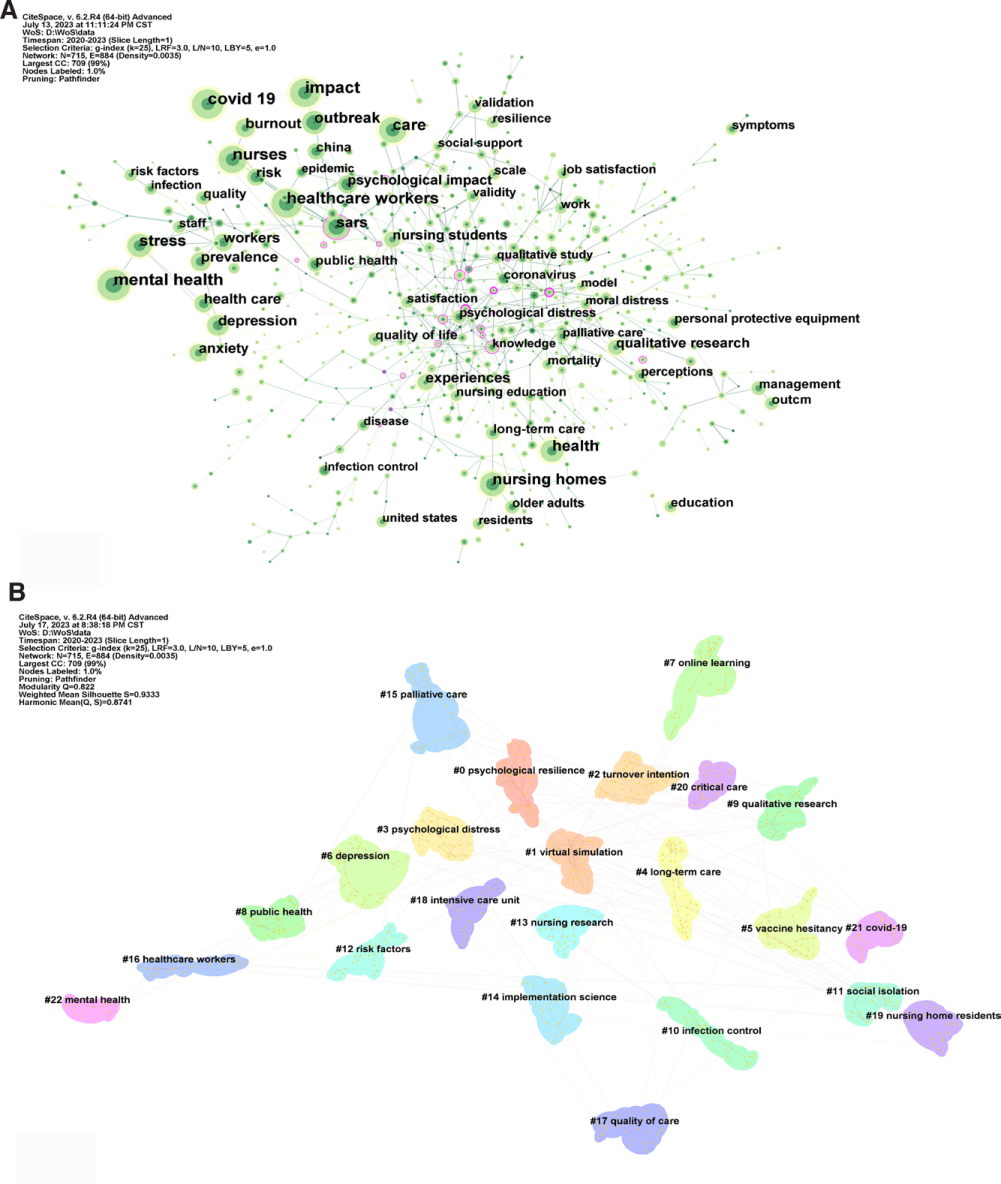
Keyword co-occurrence and clustering. (A) Network of the main keywords in publications about nursing research during the COVID-19 pandemic. (B) Keyword clusters.

Keyword co-occurrence analysis is used to address particular inquiries within a subject by examining the occurrence of keywords in close proximity to each other. Keywords can serve as a concise summary of a paper’s content, capturing its main idea in a straightforward manner. A co-occurrence network is an analytical technique based on text content. It facilitates the organization of links among various subjects within the field and enhances readers’ familiarity by examining the simultaneous occurrences of keyword pairs in a specific text. Figure 5 displays the co-occurrence findings of terms in nursing research articles pertaining to CDVID-19 and related research. In Figure 5A, the circle represents the keyword, and its size indicates the frequency of the term. A larger circle corresponds to a higher frequency. When confronted with a large number of keywords, it is challenging to categorize them into specific study subjects. However, cluster analysis can help to alleviate this issue. Figure 5B shows the grouping of the keywords using the log-likelihood rate technique. They encompass many concerns in the field of nursing research during COVID-19, including mental health (#0 psychological resilience, #2 turnover intention, #3 psychological distress, #6 depression, #22 mental health), nursing research (#1 virtual simulation, #7 online learning, #9 qualitative research, #13 nursing research), long-term care (#4 long-term care, #19 nursing home residents), policy (#5 vaccine hesitancy, #8 public health, #11 social isolation), nursing management (#10 infection control, #12 risk factors, #14 implementation science, #15 palliative care, #16 healthcare workers, #17 quality of care, #18 intensive care unit, #20 critical care), CDVID-19 (#21 covid-19).

The primary item that must be mentioned is policy (clusters #5, #8, and #11), which varies from country to country. Every country’s epidemic prevention and control policies depend on medical resources, social conditions, politics, and culture. Amidst evolving epidemic preventive and control measures, there has been a surge in nursing research papers during the COVID-19 pandemic. Most of the clusters are core components of nursing research during the COVID-19 pandemic, also known as nursing management, which focuses on effectively utilizing human and material resources to facilitate the delivery of high-quality care to patients, such as infection control (cluster #10), palliative care (cluster #15), quality of care (cluster #17), and intensive care units (cluster #18), all of which are core modalities of nursing during COVID-19 according to international guidelines.^[38,39]^ Nursing research includes virtual simulation (cluster #1), online learning (cluster #7), and qualitative research (cluster #9). Due to insufficient equipment supply, social isolation, and the redeployment of clinical instructors during the pandemic, nursing students cannot receive face-to-face clinical training.^[40]^ This discovery proves the essence of nursing education as an applied discipline, which may not be taught solely through virtual learning models.^[41]^ Therefore, researchers should pay attention to the following issues in future research: Teachers need to continuously improve their online teaching abilities and experiences and consciously encourage student-to-student, teacher-to-student, and student-to-computer interaction. In addition, emphasis should be placed on protecting students’ mental health.

Second, studies about mental health predominantly focus on frontline nurses or medical personnel. This research encompasses psychological resilience (cluster #0), psychological distress (cluster #3), and depression (cluster #6), with implications for nursing management. Nurse leaders must prioritize the mental well-being of nurses battling COVID-19 and address the causes that impact their mental health. They should provide solutions to help maintain the mental well-being of these nurses.^[42,43]^ Examples of mitigation measures include communicating orders and preventive measures from organizations or employers and providing support, including regulations, adequate insurance, compensation, counseling, and psychological support.^[44]^ In addition, knowledge of control and coping strategies,^[45]^ relatively long work experience, physical exercise,^[46]^ and social support^[47]^ can also help reduce psychological stress levels. A web-based cross-sectional survey found that healthcare workers engage in physical activities the most frequently, followed by talk therapy to minimize negative impacts on mental health.^[48]^ However, the most effective method may be to provide sufficient support for nurses, such as personal protective equipment, which should be the employer’s responsibility.

Third, nursing facilities pose a significant risk for the spread of COVID-19 among both residents and staff. Residents in nursing homes (cluster #19) face an elevated risk of experiencing severe illness and mortality owing to their advanced age and the presence of underlying medical disorders or functional impairment.^[39]^ The study found that the tragedy of nursing homes during COVID-19 was caused by decades of neglect of long-term care policies. This negligence takes several forms: lack of funding and monitoring institutions; Insufficient training and low salaries for staff; The confusion and inadequacy of medical insurance and medical subsidies in patient home care, acute post-care, and long-term care; Lack of a small-scale, high-quality model that combines home care with long-term care in nursing homes. To address the crisis in nursing homes, researchers are calling for integrating funding, policies, and new models, including institutional and noninstitutional.^[49,50]^ Implementing infection prevention and control measures can decrease the likelihood of SARS-CoV-2 transmission among nursing home patients, staff, and caregivers.^[33,51]^ Clustering using keywords provides a concise overview of the prominent topics in nursing research during the COVID-19 pandemic at the micro level, which enhances our understanding of the distribution of topics in the study during the COVID-19 pandemic.

### 3.5. Reference co-citation analysis

**Figure 6.**
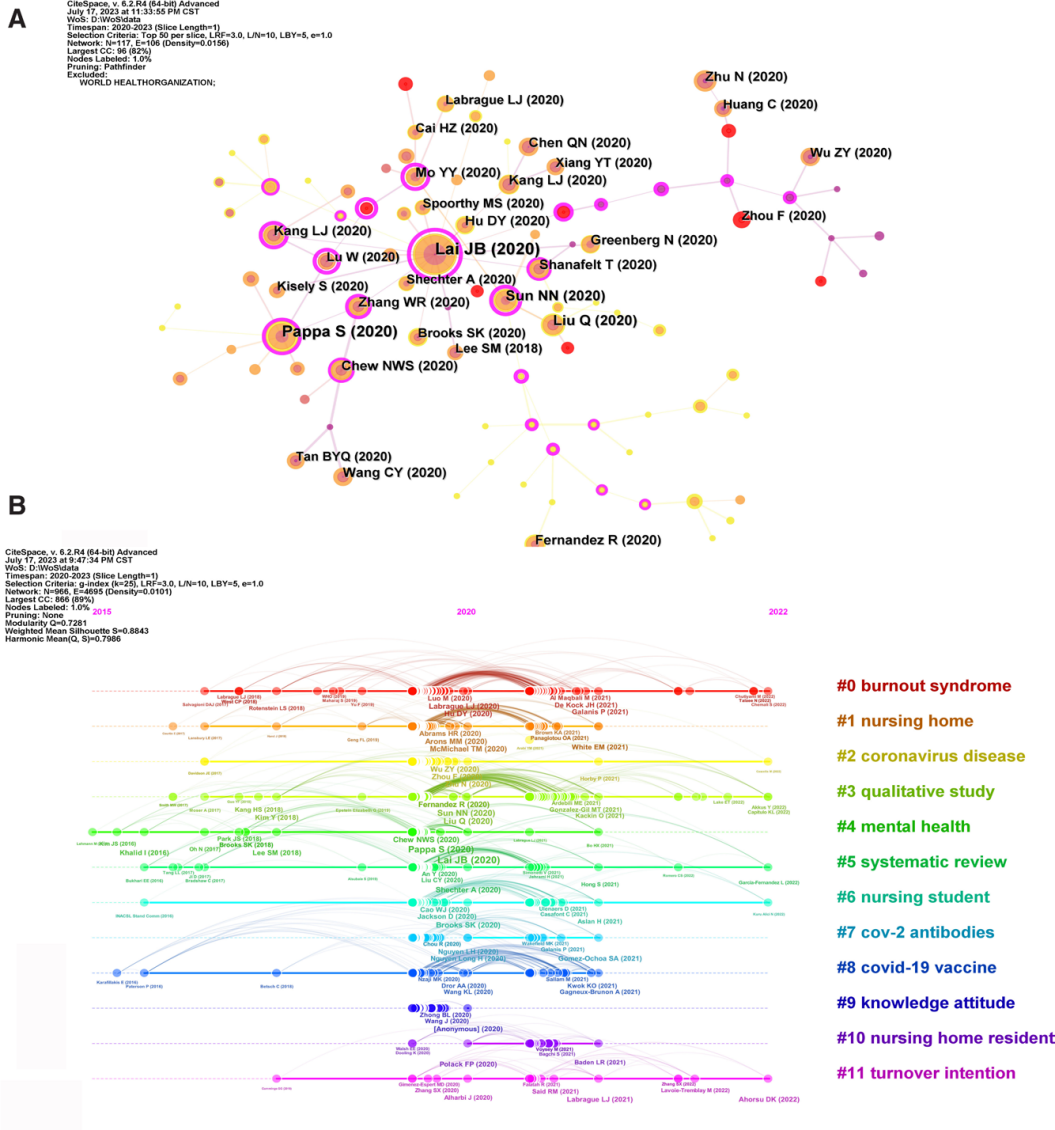
Co-citation network and timeline view of references cited by publications about nursing research during the COVID-19 pandemic. (A) References co-citation network. (B) Timeline view of references.

The concept of co-citation analysis was introduced by Small and Marshakova in 1973. It was then incorporated into the study of co-citation of references, which refers to the occurrence of 2 or more references mentioned in the literature.^[52,53]^ An analysis of the clusters and critical nodes in a co-citation network can reveal the knowledge structure of a study topic and its evolution over time. Figure 6 displays the co-citation network and chronological representation of references pertaining to nursing research during the COVID-19 pandemic and its associated investigations. In Figure 6A, the circular node represents a reference. The size of the node directly corresponds to the citation frequency of the reference, with larger nodes indicating higher citation frequency. The primary attention of researchers often focus on references that have the highest number of citations, particularly those from influential literature. Table 4 displays the 15 most frequently referenced references, providing an indication of their substance based on their titles. The major focus of these highly referenced publications is on the clinical features of COVID-19 in Wuhan, China, which can be easily identified. Wuhan is the epicenter of the initial COVID-19 pandemic and possesses crucial first-hand data, materials, and resources. Undoubtedly, it has become the central and primary subject of early publications in this discipline.^[54,55]^ Furthermore, this superior-quality literature offers crucial data and scientific proof for nursing research in the context of the COVID-19 pandemic. It establishes a convenient academic platform for researchers worldwide to engage in academic exchanges, share experiences, and showcase achievements.^[56]^ The majority of other frequently mentioned sources consist of cross-sectional or qualitative studies that examine the psychological experience or psychological burden among healthcare staff and COVID-19 patients.^[57,58]^ Additional studies are necessary to gain a deeper understanding of mental health recovery interventions for healthcare staff and patients during the COVID-19 pandemic.^[59]^

**Table 4:** Top 15 most cited references of publications about nursing research during the COVID-19 pandemic.

Rank	Article title	Year	Total cited frequency	Centrality
1	Factors associated with mental health outcomes among health care workers exposed to Coronavirus disease 2019	2020	912	0.93
2	Prevalence of depression, anxiety, and insomnia among healthcare workers during the COVID-19 pandemic: a systematic review and meta-analysis	2020	481	0.25
3	The experiences of health-care providers during the COVID-19 crisis in China: a qualitative study	2020	278	0.06
4	A qualitative study on the psychological experience of caregivers of COVID-19 patients	2020	269	0.55
5	A multinational, multicentre study on the psychological outcomes and associated physical symptoms amongst healthcare workers during COVID-19 outbreak	2020	230	0.11
6	A novel Coronavirus from patients with pneumonia in China, 2019	2020	226	0
7	Work stress among Chinese nurses to support Wuhan in fighting against COVID-19 epidemic	2020	225	0.64
8	The mental health of medical workers in Wuhan, China dealing with the 2019 novel Coronavirus	2020	211	0.08
9	Impact on mental health and perceptions of psychological care among medical and nursing staff in Wuhan during the 2019 novel Coronavirus disease outbreak: a cross-sectional study	2020	207	0.5
10	Implications for COVID-19: a systematic review of nurses’ experiences of working in acute care hospital settings during a respiratory pandemic	2020	203	0
11	Managing mental health challenges faced by healthcare workers during COVID-19 pandemic	2020	199	0
12	Mental health care for medical staff in China during the COVID-19 outbreak	2020	197	0
13	Understanding and addressing sources of anxiety among health care professionals during the COVID-19 pandemic	2020	197	0.11
14	Mental health and psychosocial problems of medical health workers during the COVID-19 epidemic in China	2020	188	0.25
15	Epidemiology of COVID-19 in a long-term care facility in King County, Washington	2020	186	0

Three nodes stand out in the network, suggesting that they can provide valuable insights into the key factors influencing the progress of nursing research during the COVID-19 pandemic. The first noteworthy event occurred in 2020. A cross-sectional study examined the significant percentage of Chinese healthcare workers who reported symptoms of depression, anxiety, insomnia, and distress. This is particularly true for women, nurses, individuals in Wuhan, and frontline healthcare workers directly involved in diagnosing, treating, or providing nursing care to patients with suspected or confirmed COVID-19.^[60]^ The study’s findings indicate that healthcare professionals exposed to COVID-19 face a significant likelihood of experiencing adverse mental health consequences. Therefore, it is crucial to provide psychological support and therapies. This essay offers an essential perspective on the need to prioritize the mental well-being of healthcare professionals, whether it is for their long-term ability to perform their job or for their immediate reaction to the crisis caused by the COVID-19 pandemic.^[61]^

The following key milestones occurred in 2020. A previous study sought to compile and examine current information about the frequency of sadness, anxiety, and sleeplessness among healthcare professionals’ well-being during the COVID-19 pandemic. The COVID-19 pandemic has the capacity to profoundly impact the emotional well-being of healthcare professionals who are at the forefront of this disaster. Hence, it is crucial to promptly observe the levels of mood, sleep, and other mental health problems to comprehend the elements that influence them and provide customized therapies.^[62]^ Overall, this study reinforces this notion more extensively and methodically than the initial major point, offering more thorough recommendations for the mental well-being of nursing research throughout the COVID-19 pandemic. The latter promptly documented the accounts of physicians and nurses who lacked knowledge of infectious diseases and were enlisted to deliver medical care to COVID-19 patients during the initial phase of the COVID-19 epidemic in Hubei, China.^[56]^ Nursing research has been intricately linked to the emergence and progression of COVID-19. The study’s results indicate that effective nursing measures should be founded upon prompt intervention as well as scientifically and practically sound therapy. These 3 salient occasions might be regarded as pivotal occurrences in nursing research during the COVID-19 pandemic and exert a deep influence on it.

CiteSpace provides a distinctive chronological perspective to examine the research frontiers, knowledge base, and duration, as well as the literature that has significantly contributed to their development. The timeline view is a visualization tool that combines clustering and time-slicing methods. The ranking of items is determined based on their timing of arrival, either early or late, following the clustering. This ranking shows the distribution of themes within the field and illustrates the trends and linkages between the study subjects over time. Figure 6B displays a timeline at the top, where each node represents the publishing year. The oldest date was from 2015 onward. All references belonging to the cluster are represented by a straight line at the same horizontal position. The cluster label is positioned at the far-right end of the line. The interconnected network was partitioned into co-citation clusters of references. The references mentioned above are research fronts linked to these clusters. Each cluster reflects essential knowledge of the underlying expertise. Figure 6B demonstrates that the COVID-19 pandemic is a newly developing area of research, with all included materials being published within the last 3 years. Consequently, it is not feasible to utilize the timeline view function of CiteSpace to depict its evolving trends. COVID-19 is recognized as a rapidly spreading infectious illness. Nursing research is now focused on the occurrence and progression of COVID-19, particularly in relation to clusters #0 and #2. As more concrete information was gathered, national policies and organizations provided technical directions based on empirical backing from nursing research.^[19]^

Although there is much data supporting the effectiveness of mass vaccination as a feasible strategy to effectively manage the local outbreak and worldwide transmission of the COVID-19 pandemic, the reluctance to receive vaccines continues to hinder the achievement of total population inoculation against highly contagious illnesses. Healthcare providers who have the closest contact with the vaccinated population should gather pertinent information and data both before and after vaccination (clusters #7 and #8). Simultaneous with the swift advancements in COVID-19 vaccinations on a worldwide scale, apprehensions over the safety of the vaccine may add to vaccine hesitancy. There is an urgent need for educational initiatives that specifically target individuals at risk for vaccine reluctance. These campaigns aimed to counteract misinformation and prevent low vaccination.^[63]^

The current focal points of discussion (clusters #1 and #10) are key elements in enhancing healthcare quality during the COVID-19 pandemic. The findings examined the transmission of COVID-19 and analyzed the effectiveness of symptom-based screening in nursing homes. They strongly confirmed that infection control measures that only targeted symptomatic residents were not sufficient to prevent transmission.^[64]^ The swift dissemination of COVID-19 and the gravity of symptoms that can be induced in a subset of infected persons has significantly strained healthcare systems. Simultaneously, healthcare professionals navigate the challenges of social change and emotional strain experienced by everyone. However, they also encounter heightened vulnerability to exposure, excessive workloads, ethical issues, and a rapidly changing practice environment that deviates dramatically from their accustomed norms.^[65]^ The COVID-19 pandemic has brought forth a significant problem of occupational burnout among nurses. It is imperative to train nurses to handle the challenges posed by the COVID-19 pandemic effectively. Identifying the risk factors for burnout might provide nurses and healthcare systems with a valuable tool to effectively respond to future waves of COVID-19.^[66]^ The empirical literature on nursing during the COVID-19 outbreak provides a reference for healthcare workers dealing with COVID-19. More comprehensive research should be conducted (clusters #4, #6, #9, and #11). The contribution of qualitative studies (clusters #3 and #5) to nursing theory and knowledge is increasing, particularly in exploring new topics. Systematic reviews can provide vital information for healthcare workers as a source of high-quality evidence in evidence-based medicine.^[56,67]^ Concisely, the co-citation analysis of nursing research pertaining to the COVID-19 pandemic offers useful insights into the development of knowledge structures and shifts in research focal points. This aids in identifying the central subject and crucial emphasis within this field.

## 4. Conclusions

The COVID-19 pandemic had a substantial impact on people, families, communities, and cultures globally during the PHEIC. A significant number of COVID-19 patients, who may require high-quality clinical treatment, impose a significant strain on health systems and healthcare staff. Despite variations in research platforms, development procedures, timetables, and coordination concerns, researchers universally agree on the pressing necessity to conduct research and produce medical countermeasures such as vaccinations, medicines, diagnostics, and nursing. The visual analytic technique we employed in this review actively enhances traditional review and survey papers, and is invaluable for identifying significant advancements in the extensive body of published studies.

Our study of the literature on nursing during the COVID-19 pandemic and associated studies over the last 3 years indicates that nursing during this period has seen rapid development. Nevertheless, there is room for improvement to achieve a more equitable distribution of development across different regions and nations. Several countries, including the United States, China, and the UK, have established systematic and standardized nursing models along with well-developed nursing research systems. However, in many low-income and middle-income countries and regions, there is a lack of awareness and participation in nursing research during the COVID-19 pandemic. Consequently, these nations have witnessed the establishment of several highly engaged academic communities focused on nursing research throughout the COVID-19 pandemic. The University of London, Harvard University, and University of California System are the most prominent academic institutions. Nevertheless, the level of engagement and collaboration in the field of nursing during the COVID-19 pandemic is inadequate across prominent nations and academic institutions. To ensure a well-rounded and extensive advancement of nursing worldwide amid the COVID-19 pandemic, it is imperative to enhance transnational and cross-team collaboration, necessitating the involvement of additional international organizations specializing in nursing throughout this crisis. We anticipate the occurrence of interdisciplinary and inter center collaboration, as it would expedite and enhance the contribution of worldwide COVID-19 nursing research, ultimately leading to an improvement in patients’ quality of life. Furthermore, nursing in the midst of the COVID-19 pandemic is characterized by multiple disciplines. The advancement and expansion of nursing research necessitates the coordinated development and mutual integration of clinical medicine, essential medicine, psychology, public health management, and telematics science. This implies that there is a need to prioritize the establishment of a COVID-19 nursing care team consisting of professionals from many disciplines. Initial nursing research conducted during the COVID-19 pandemic mostly concentrated on nursing administration, which efficiently leveraged people and material resources to support the provision of exceptional care to patients. The following studies seek to combine and examine the fact that frontline nurses exposed to COVID-19 face a significant likelihood of acquiring mental health issues and require psychological treatment or interventions. National policy responses and organizations provide technical recommendations based on empirical evidence from nursing research. The reluctance to receive vaccines continues to impede full immunization coverage for highly contagious illnesses. Healthcare providers have the most direct interaction with the vaccinated population, making it simple to gather pertinent information and data, both before and after vaccination. The findings examined the spread of COVID-19 and analyzed the effectiveness of symptom-based screening in nursing homes.

To summarize, our study has provided valuable insights into acquiring knowledge on nursing research during the COVID-19 pandemic, pinpointed possible partners for researchers with an interest in nursing, and uncovered prevailing research patterns and popular subjects. Simultaneously, our efforts will benefit a greater number of healthcare professionals and individuals affected by COVID-19, particularly in underdeveloped nations and areas where nursing research is lacking. Gaining a more profound comprehension of nursing research amid the COVID-19 pandemic has the potential to stimulate curiosity in this field, thereby enhancing the care and prognosis of the vast global population afflicted by COVID-19.

## 5. Limitation and future research

The following are some intrinsic limitations of this graphical and bibliometric analysis: The research exclusively utilized datasets published in English, deliberately excluding papers that employed the Chinese language. Consequently, the study findings may be impacted by the need for prestigious journals from countries where English is not the primary language as well as the constraints imposed by the available data sources. Furthermore, this analysis should also consider the impact of recently published studies. Future studies should use alternative analytical approaches to uncover the latent worth of recently released publications. Furthermore, this study exclusively incorporated data acquired from the Web of Science while disregarding the data obtained from PubMed. Owing to its open-access nature, gathering PubMed data might offer more benefits and perhaps reveal distinct perspectives. Hence, it is advisable for future researchers to gather data from many sources, particularly from PubMed, to conduct a more comprehensive analysis of this idea.

## Acknowledgments

We express our gratitude to Professor Chaomei Chen for his development of CiteSpace and for his decision to make it accessible to the public.

## Author contributions

**Conceptualization:** Lu Yang.

**Data curation:** Lu Yang, Xin Mu.

**Formal analysis:** Lu Yang.

**Funding acquisition:** Yanbiao Liao.

**Investigation:** Lu Yang.

**Methodology:** Lu Yang.

**Project administration:** Yanbiao Liao.

**Software:** Lu Yang, Yanbiao Liao.

**Supervision:** Xin Mu.

**Validation:** Yao Wang.

**Visualization:** Yao Wang.

**Writing – original draft:** Lu Yang.

**Writing – review & editing:** Lu Yang.
